# Calibration and validation of the Angstrom–Prescott model in solar radiation estimation using optimization algorithms

**DOI:** 10.1038/s41598-022-08744-6

**Published:** 2022-03-22

**Authors:** Seyedeh Nafiseh Banihashemi Dehkordi, Bahram Bakhtiari, Kourosh Qaderi, Mohammad Mehdi Ahmadi

**Affiliations:** Department of Water Engineering, Shahid Bahonar University of Kerman, Kerman, Iran

**Keywords:** Solar energy, Engineering

## Abstract

The Angstrom–Prescott (A–P) model is widely suggested for estimating solar radiation (R_s_) in areas without measured or deficiency of data. The aim of this research was calibration and validation of the coefficients of the A–P model at six meteorological stations across arid and semi-arid regions of Iran. This model has improved by adding the air temperature and relative humidity terms. Besides, the coefficients of the A–P model and improved models have calibrated using some optimization algorithms including Harmony Search (HS) and Shuffled Complex Evolution (SCE). Performance indices, i.e., Root Mean Square Error (RMSE), Mean Bias Error, and coefficient of determination (R^2^) have used to analyze the models ability in estimating R_s_. The results indicated that the performance of the A–P model had more precision and less error than improved models in all the stations. In addition, the best results have obtained for the A–P model with the SCE algorithm. The RMSE varies between 0.82 and 2.67 MJ m^−2^ day^−1^ for the A–P model with the SCE algorithm in the calibration phase. In the SCE algorithm, the values of RMSE had decreased about 4% and 7% for Mashhad and Kerman stations in the calibration phase compared to the HS algorithm, respectively.

## Introduction

The solar radiation (R_s_) received from the Earth’s surface is one of the most important factors affecting the thermal balance of the atmospheric-Earth system. The R_s_ precise measurement or estimation has been required for accurate design and management in irrigation and water resource planning and management, agriculture, meteorology, climatology, energy engineering, solar energy systems, and especially in hydrology^1,2^. One significant part of the hydrological cycle is the evapotranspiration (ET) process that is widely used for agricultural, irrigation management, and water resources planning^3^. The R_s_ is the primary input variable in the calculation of ET^4^. Concerning to the cost and the maintenance and calibration requirements of the R_s_ estimating instrument, missing data, or due to instrument failure or other related problems, it might be that the estimates of R_s_ are not available in several regions^5^. For this reason, several methods have been presented to estimate R_s_ based on different types of methods such as satellite remote sensing^6,7^, machine learning^8–10^, numerical, and artificial intelligence^11,12^. Guermoui et al.^13^ used two Support Vector Machine (SVM) models for estimating global solar radiation in Algeria. There are some complexes and difficulties in using these methods for R_s_ estimation such as: requiring many input variables, large datasets, coarse spatial resolution, and the final model may not apply to other areas. Besides, there is no satellite-based database to cover the study areas^14^.

Another kind of method that has been developed and widely used for estimating R_s_ are empirical models^15^. These models based on meteorological variables are a substitute to estimate R_s_. Besides, these models using the easily accessible meteorological variables, such as sunshine duration, maximum and minimum air temperatures (T_max_, T_min_), cloudiness, relative humidity (RH), and precipitation, are attractive for their plainness, efficiency, and lower data requirement^16^. More previous research has determined that the sunshine-based models consistently outperform other types of models^17,18^. These models do not require many input variables, but their coefficients should be calibrating based on region and input data. However, the requirements to calibrate empirical models demonstrate that their coefficients are changing with locations. The station-dependent coefficients limit the regional application of the empirical models, which is a big challenge for spatial rasterization. The model coefficients for the regional usage must calibrate in order to solve this problem.

Many models have developed for estimating R_s_. One of the most famous empirical sunshine-based models is the Angstrom–Prescott (A–P) model. The A–P model has applied to estimate global solar radiation based on measured sunshine hours. This model is widely used for its simpleness and remarkable performance^19,20^. One of the original constraints of the A–P model is that it requires calibration using local estimated R_s_ data. Where no measured values for global solar radiation are available in some stations, Angstrom prospered values of 0.2, 0.5, and Prescott 0.22, and 0.54 for the empirical coefficients ‘a’ and ‘b’, respectively^21^. Given its simpleness and premiere performance compared with other empirical models, its reference values for radiation coefficients ‘a’ and ‘b’, given by the Food and Agriculture Organization (FAO) Irrigation and Drainage Paper No. 56 (FAO56: a = 0.25, b = 0.5), can be used in cases where R_s_ data are not available^16,22^. FAO56 proposed the A–P model, which is a simple method to estimate the daily global solar radiation. The results of previous research showed that the application of the FAO pre-defined the A–P coefficients, for a variety of climatic and geographical conditions (regardless of climate effect) could challenge the validity of the FAO56-PM method^23^. Therefore, many researchers performed a temporal and spatial calibration of ‘a’ and ‘b’^24^. On the other hand, researchers have attempted to estimate R_s_ in addition to the sunshine, take advantage of other variables such as air temperature, relative humidity, cloudiness, saturation vapor pressure, and even precipitation.

Recently many kinds of meta-heuristic algorithms have used to calibrate a different type empirical model in the real problem. Few usages of metaheuristic methods to solve solar energy problems have reported; the Genetic Algorithm (GA) is one of these methods. Sen et al.^25^ have used GA for the designation of the A–P model coefficients.

Harmony Search (HS) is one of the well-known and influential optimization algorithms^26^, which emulates the music extemporization process where musicians extemporize their instruments’ pitches searching for a perfect state of harmony, was developed by Geem et al.^27^. The HS algorithm has been recently applied to different engineering optimization problems including optimized design of water dispensation network^28^, optimal performance of a multi-reservoir system for hydropower and irrigation^29^, simulation of irrigation systems^30^, an optimization model for groundwater management objectives^31^, and recognition of unknown groundwater pollution sources^32^. To fix the defects of the HS algorithm, the methods such as the Global Harmony Search (GHS) and Improved Harmony Search (IHS) algorithm developed. Another optimization algorithm used for effective global minimization and calibration of hydrologic models is the Shuffled Complex Evolution (SCE) algorithm^33^. In addition, this algorithm has been used widely for the calibration of different rainfall-runoff models^34,35^, for the rehabilitation of water distribution networks^36^, and optimizing urban water supply Headwork systems^37^.

There has not been much research on computing R_s_ by optimization algorithms in Iran, and only one research conducted in Mashhad^26^ examined. This is the first research by optimization algorithms to calibrate the A–P model coefficients in Iran. Through these algorithms, the A–P model coefficients have calibrated faster and more accurately, and R_s_ is a fundamental input for calculating ET^38^, have estimated more correctly. Accurate estimation of R_s_ provides an accurate calculation of ET. The exact calculation of ET is necessary for many applications, such as improving water usage, agricultural planning, and effective water resources management, especially in arid and semi-arid climates.

This research aims to calibrate and improve the A–P model for estimating R_s_ at six meteorological stations in arid and semi-arid climates of Iran using optimization algorithms including HS, IHS, GHS, and SCE. Then to investigate the effect of T and RH variables on the efficiency of the A–P model to estimate R_s_, three improved A–P models were developed by adding terms of T_max_, T_min_, and mean relative humidity (RH_mean_) and calibrated using applied optimization algorithms.

## Material and methods

### Study area

**Figure 1:**
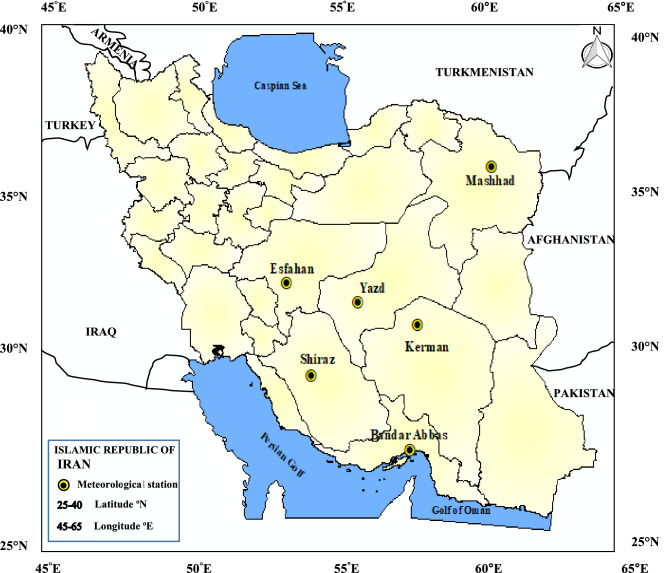
Location of meteorological stations.

Iran is situated among latitudes of 25°N to 40°N and longitudes of 46°E to 65°E with an area of 1,648,000-km^2^. Most parts of Iran are arid and semi-arid climates. On the other hand, low irrigation efficiency in agricultural fields requires that the amount of ET and water requirement of plants that require an accurate estimate of R_s_ has calculated. In this research six meteorological stations, which situated at arid and semi-arid climates of Iran, have selected to evaluate the performance of the calibrated A–P model in R_s_ estimation. The selected stations have arid and semi-arid climates based on the De Martonne climate classification method^39,40^ from 1992 to 2017 and reliable long-term data (Fig. 1). The criteria for selecting the meteorological stations have based on the climate sort and the availability of the measured R_s_.

### Data and quality control

Daily meteorological data from six radiation stations have obtained from the Islamic Republic of Iran Meteorological Organization (IRIMO). The geographic and meteorological characteristics of the studied stations have presented in Table 1. In this research, the following meteorological characteristics have used as the inputs of the A–P and the three improved models: T_max_, T_min_, RH_mean_, and R_s_ (MJ m^−2^ day^−1^), maximum possible daily duration of sunshine hours (N), and mean the daily number of sunshine duration (n). Due to the importance of radiation data, the quality control of the observed daily global R_s_ was carried^41^:

- If either the fluency index (R_s_/R_a_) or relative sunshine hours (n/N) were greater than one, the data for that day were deleted from the dataset.
- If R_s_ was greater than 0.78 × R_a_, the data for that day have deleted from the dataset.
- If R_s_ was lower than 0.03 × R_a_, the data for that day have deleted.
- If there were ten or more days of lost data in the same month, the data for that month has omitted.

**Table 1:** Geographical and meteorological characteristics for the studied stations.

Station	Bandar Abbas	Esfahan	Kerman	Mashhad	Shiraz	Yazd
Lat. (°N)	27.19	32.46	30.15	36.16	29.53	31.88
Lon. (°E)	56.3	51.6	56.5	59.38	52.58	54.35
Elev. (M)	17	159	175	999	1486	1222
Maximum temperature (°C)	47	43	41.4	43.4	42.4	45.6
Minimum temperature (°C)	2.6	− 19.3	− 23.2	− 21.37	− 9	− 6.7
Average sunshine (H)	8.44	8.6	8.17	7.27	8.96	8.94
Average R_s_ (MJ M^−2^ Day^−1^)	19.01	16.74	18.77	16.24	19.78	19.46
RH (%)	63.40	35.92	38.4	53.98	40.54	28.81
Calibration period	1992–2012	1992–2012	1992–2012	1992–2012	1992–2012	1992–2012
Climate	Semi-arid	Arid	Arid	Semi-arid	Semi-arid	Arid
Validation period	2013–2017	2013–2017	2013–2017	2013–2017	2013–2017	2013–2017

### Models and optimization algorithms

#### Models

The A–P model has based on sunshine, and to examine the effect of other meteorological variables, the following models presented have examined in Table 2.

**Table 2:** Improved A–P model based on terms of T_max_, T_min,_ and RH_mean_.

Models			Coefficients
Model 1	Include air temperature	R_s_ = [a_1_ + b_1_(n/N) + c(T_max_ − T_min_)] × R_a_	a_1_, b_1_, c
Model 2	Include relative humidity	R_s_ = [a_2_ + b_2_(n/N) + d(RH_mean_)] × R_a_	a_2_, b_2_, d
Model 3	Combined Model 1 and Model 2	R_s_ = [a_3_ + b_3_(n/N) + c_1_(T_max_ − T_min_) + d_1_(RH_mean_)] × R_a_	a_3_, b_3_, c_1_, d_1_

### Optimization algorithm

The optimization algorithms have coded with MATLAB R2018a (9.4.0.813654). These algorithms have applied to find the optimal solution to a given calculational problem that minimizes or maximizes a special function. In this research, optimization algorithms including SCE, IHS, GHS, and HS have used.

#### Shuffled Complex Evolution (SCE) algorithm

The SCE algorithm has expanded at the University of Arizona^42^. Its strategy combines the strengths of the controlled random search (CRS) algorithms with the concept of competitive evolution^43^ and the newly modified concept of complex shuffling. The most important steps of the SCE have displayed in Algorithm 1.
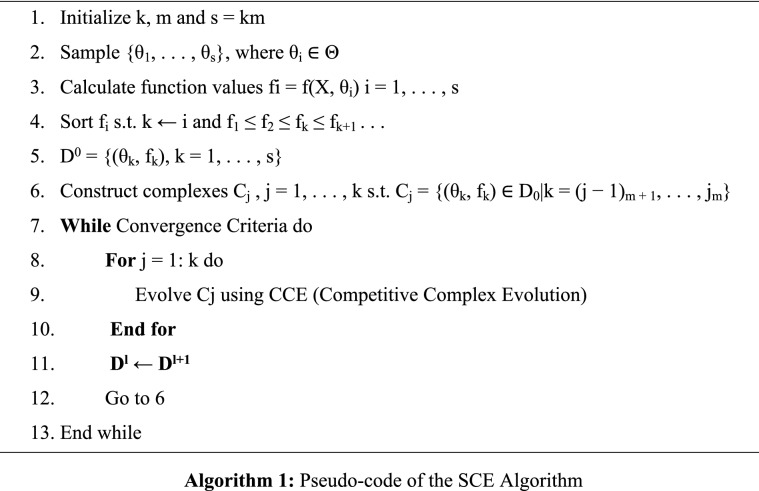


#### Harmony Search (HS) algorithm

When listening to a beautiful piece of classical music, who has ever wondered if there is any connector between music and finding an optimal solution to a tough design problem such as the water distribution networks or other design problems in engineering? For the first time, scientists have found such a fascinating connection by expanding a new algorithm, called HS. Geem et al. first expanded the HS in 2001.

1 $${\text{HM}} = \left[ {\begin{array}{*{20}l} {{\text{x}}_{{{11}}} } \hfill & {{\text{x}}_{{{12}}} } \hfill & {{\text{x}}_{{{13}}} } \hfill & \cdots \hfill & {{\text{x}}_{{{\text{1n}}}} } \hfill \\ {{\text{x}}_{{{21}}} } \hfill & {{\text{x}}_{{{22}}} } \hfill & {{\text{x}}_{{{23}}} } \hfill & \cdots \hfill & {{\text{x}}_{{{\text{2n}}}} } \hfill \\ \vdots \hfill & \vdots \hfill & \vdots \hfill & \vdots \hfill & \vdots \hfill \\ {{\text{x}}_{{{\text{HMS1}}}} } \hfill & {{\text{x}}_{{{\text{HMS2}}}} } \hfill & {{\text{x}}_{{{\text{HMS3}}}} } \hfill & \cdots \hfill & {{\text{x}}_{{{\text{HMSn}}}} } \hfill \\ \end{array} } \right]$$

Harmony memory considering (HMC) rule:

- For this rule, a new random number r_1_ has produced within the range [0, 1].
- If r_1_ < HMCR, where HMCR is the harmony memory consideration rate, then the first decision variable in the new vector x_ij_^new^ is elected randomly from the values in the present HM as follows:
  2 $${\text{x}}_{{{\text{ij}}}}^{{{\text{new}}}} {\text{ = x}}_{{{\text{ij}}}} {,}\,\,\,{\text{x}}_{{{\text{ij}}}} \in \left\{ {\begin{array}{*{20}l} {{\text{x}}_{{{\text{1j}}}} {,}} \hfill & {{\text{x}}_{{{\text{2j}}}} {,}} \hfill & {{\text{x}}_{{{\text{3j}}}} {,}} \hfill & { \cdots ,} \hfill & {{\text{x}}_{{{\text{HMSj}}}} } \hfill \\ \end{array} } \right\}$$

The most important steps of the HS have displayed in Algorithm 2.
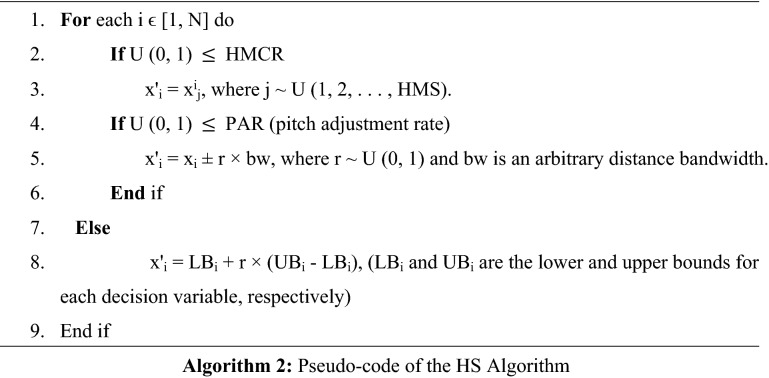


#### Developed Harmony Search (HS) algorithm

The HS is good at recognizing high-performance areas of the solution space in a sensible amount of time but it gets difficult to do a local search for numeral usages. To improve the exact situation feature HS algorithm, IHS and GHS use a new method that increases the precision setting and the convergence rate of HS. The IHS usages a new method to generate new solution vectors that increase the precision and convergence rate of the HS. Omran and Mahdavi^44^ suggested a new variation of HS, called GHS. First, in GHS, a dynamically updating scheme of parameter PAR usage in IHS^45^ employed to improve the performance of GHS. Second, GHS modifies the pitch adjustment step of HS to use the best harmonic guidance information in harmony memory (HM). In the altered stage, GHS not only destroys the parameter bandwidth (BW), which is difficult to set because it can take any values in the range of [0,$\infty$] but also introduces a social term of the best harmony with HS. These two methods (IHS, GHS) have developed to overcome the disadvantages of the original method.

### Methodology

One of the most popular empirical sunshine-based models is the A–P model. This model has used to estimate global solar radiation based on measured sunshine hours. The model is as follows^46,47^:

3 $${\text{R}}_{{\text{s}}} = {\text{R}}_{{\text{a}}} \left[ {{\text{a}} + {\text{b}}\left( \frac{n}{N} \right)} \right]$$

Here R_s_ and R_a_ is daily global solar radiation and daily extraterrestrial solar radiation (MJ m^−2^ day^−1^), respectively R_a_ , n is the mean daily number of sunshine duration (h), N is the maximum possible daily duration of sunshine hours (h) and ‘a’ and ‘b’ are empirical coefficients which must be calibrated based on long-term measured R_s_ data. R_a_ data for each day and location have gained from the estimation of geographical parameters including solar declination, solar constant, and the time of the year as shown in the method below^48^:

4 $${\text{R}}_{{\text{a}}} = {37}.{\text{6d}}_{{\text{r}}} \left[ {\upomega _{{\text{s}}} \sin \emptyset {\text{sin}}\updelta + {\text{cos}}\emptyset {\text{cos}}\updelta {\text{sin}}\upomega _{{\text{s}}} } \right]$$

Here d_r_ is the eccentricity correction factor of the Earth’s orbit (Eq. 5); ω_s_ is the sunshine hour angle of the sun at sunrise in radians (Eq. 6), ϕ is the latitude of the station, and δ is the solar declination angle in radians Eq. (7):

5 $${\text{d}}_{{\text{r}}} = 1 + 0.033\cos \left( {{\text{J}}_{{\text{s}}} \frac{360}{{365}}} \right)$$

6 $$\upomega _{{\text{s}}} = \arccos ( - \tan \emptyset \tan\updelta )$$

7 $$\updelta = 0.409\sin \left( {\frac{360}{{365}}{\text{J}}_{{\text{s}}} - 1.39} \right)$$

The maximum possible average daily length of sunshine hour N can calculate by Duffie–Beckman 1991 model:

8 $${\text{N}} = \frac{2}{15}\upomega _{{\text{s}}}$$

### Performance indicators

The performance indicators discussed in this research were the coefficient of determination (R^2^), Mean Bias Error {MBE (MJ m^−2^ day^−1^)}, Root Mean Square Error {RMSE (MJ m^−2^ day^−1^)}. These indicators calculated as follows:

9 $${\text{R}}^{2} = \left[ {\frac{{\mathop \sum \nolimits_{{{\text{i}} = 1}}^{{\text{m}}} \left( {{\text{R}}_{{{\text{estim}}}} -\upmu _{{{\text{estim}}}} } \right)\left( {{\text{R}}_{{{\text{meas}}}} -\upmu _{{{\text{meas}}}} } \right)}}{{\left[ {\mathop \sum \nolimits_{{{\text{i}} = 1}}^{{\text{m}}} \left( {{\text{R}}_{{{\text{estim}}}} -\upmu _{{{\text{estim}}}} } \right)^{2} } \right]^{0.5} \left[ {\mathop \sum \nolimits_{{{\text{i}} = 1}}^{{\text{m}}} \left( {{\text{R}}_{{{\text{meas}}}} -\upmu _{{{\text{meas}}}} } \right)^{2} } \right]^{0.5} }}} \right]^{2}$$

10 $${\text{RMSE}} = \left[ {\frac{{1}}{{\text{M}}}\sum\nolimits_{{\text{i = 1}}}^{{\text{M}}} {({\text{R}}_{{{\text{estim}}}} - {\text{R}}_{{{\text{meas}}}} )^{2} } } \right]^{1/2}$$

11 $${\text{MBE}} = \frac{{1}}{{\text{M}}}\sum\nolimits_{{\text{i = 1}}}^{{\text{M}}} {({\text{R}}_{{{\text{estim}}}} - {\text{R}}_{{{\text{meas}}}} )}$$

Here M is the total number of estimated values, R_estim_ and R_meas_ are, estimated and measured daily global solar radiation values respectively, μ_estim_ is the average of the daily estimated values and μ_meas_ is the average of the daily measured values. The R^2^ stands for the proportion of variability in a data set that has calculated by the model. The MBE, RMSE, and the R^2^ statistical indices have used to evaluate the performance of applied optimization methods and improved the A–P model for R_s_ estimating. The negative values of MBE represent the difference between the estimated data and measured data. If the MBE value is positive, then the estimated values are overestimated and if the MBE value is negative, it means underestimating the estimated values. Whatever the MBE value is closer to zero indicates the accuracy of the model and the closeness of the amount of estimation data to the measured data.

## Results and discussion

The calibrated coefficients for the A–P model and the models obtained with different optimization algorithms, the empirical coefficients (a, b, c, d) for four models, and the RMSE, R^2^, MBE values are shown in Tables 3 and 5 respectively.

**Table 3:** The locally calibrated of the models coefficients for the selected stations using optimization algorithms.

Station	Algorithm	A–P Model		Model 1			Model 2			Model 3			
		a	b	a_1_	b_1_	c	a_2_	b_2_	d	a_3_	b_3_	c_1_	d_1_
Bandar Abbas	SCE	0.38	0.35	0.39	0.31	− 0.0015	0.38	0.35	0	0.4	0.35	− 0.0019	0
	HS	0.38	0.36	0.36	0.35	0.0036	0.47	0.33	− 0.0012	0.3	0.38	0.0078	0.0002
	IHS	0.39	0.33	0.39	0.36	− 0.0046	0.32	0.37	0.0008	0.29	0.33	0.0058	0.0012
	GHS	0.36	0.37	0.35	0.39	− 0.0006	0.39	0.35	− 0.0002	0.47	0.20	− 0.0016	− 0.0003
Esfahan	SCE	0.15	0.58	0.15	0.58	− 0.0004	0.15	0.58	0	0.15	0.58	− 0.0004	0
	HS	0.13	0.60	0.18	0.60	− 0.0076	0.20	0.54	− 0.0007	0.1	0.54	0.0152	− 0.0008
	IHS	0.16	0.56	0.12	0.64	− 0.0021	0.16	0.54	0.0005	0.15	0.57	− 0.0006	0.0003
	GHS	0.15	0.57	0.14	0.59	0	0.13	0.59	0	0.12	0.63	0	0
Kerman	SCE	0.27	0.51	0.27	0.51	− 0.0013	0.28	0.49	− 0.0003	0.29	0.50	− 0.0019	− 0.0003
	HS	0.28	0.47	0.21	0.44	0.0109	0.18	0.59	0.0015	0.34	0.57	− 0.0058	− 0.0022
	IHS	0.24	0.54	0.32	0.46	− 0.0025	0.38	0.41	− 0.0012	0.19	0.58	− 0.0011	0.0006
	GHS	0.26	0.50	0.28	0.50	− 0.0013	0.30	0.48	− 0.0006	0.36	0.50	− 0.0061	− 0.0009
Mashhad	SCE	0.22	0.62	0.22	0.62	− 0.0001	0.23	0.61	0	0.23	0.61	− 0.0007	− 0.0001
	HS	0.24	0.59	0.19	0.56	0.01	0.12	0.65	0.0014	0.23	0.58	0.0077	− 0.0008
	IHS	0.23	0.61	0.26	0.63	− 0.0074	0.29	0.58	− 0.0008	0.30	0.61	− 0.0016	− 0.0013
	GHS	0.21	0.63	0.25	0.63	− 0.0055	0.23	0.59	0	0.26	0.61	− 0.0002	− 0.0008
Shiraz	SCE	0.25	0.53	0.24	0.53	0.0003	0.29	0.51	− 0.0006	0.30	0.51	− 0.0012	− 0.0007
	HS	0.26	0.50	0.11	0.51	0.0029	0.18	0.57	0.0009	0.40	0.52	− 0.0064	− 0.0023
	IHS	0.27	0.51	0.3	0.51	− 0.0029	0.35	0.49	− 0.0017	0.18	0.52	0.0107	− 0.0002
	GHS	0.20	0.58	0.24	0.55	− 0.0002	0.23	0.58	− 0.0004	0.37	0.46	− 0.0063	− 0.0007
Yazd	SCE	0.18	0.53	0.19	0.53	0.0003	0.22	0.64	− 0.0006	0.24	0.65	− 0.0035	− 0.0007
	HS	0.20	0.64	0.31	0.62	− 0.0117	0.16	0.69	0.0003	0.10	0.63	0.015	0
	IHS	0.19	0.66	0.16	0.66	0.0034	0.21	0.68	− 0.0016	0.28	0.52	0.0084	− 0.0015
	GHS	0.18	0.67	0.17	0.69	− 0.0021	0.26	0.60	− 0.0007	0.35	0.58	− 0.0075	− 0.0015

The statistics of the calibrated A–P coefficients in six meteorological stations (Table 3) showed that the coefficient ‘a’ had low values in Esfahan in the HS algorithm and high values in Bandar Abbas in the IHS algorithm. The coefficients ‘a’ and ‘b’ predicted by four models and by four optimization algorithms. Adding T_max_, T_min_, and RH_mean_ terms to the A–P model have had little effect on improving the radiation estimation used by the models. Zero or near-zero values of T_max_, T_min_, and RH_mean_ coefficients indicate this.

Statistical analysis (kurtosis, Skewness) on data shown that Table 4. In this table, Skewness essentially measures the symmetry of the distribution, while Kurtosis determines the heaviness of the distribution tails. In positively Skewness, the mean of the data is greater than the median.

**Table 4:** Statistical analysis (Kurtosis, Slowness) on data.

Station	Bandar Abbas	Esfahan	Kerman	Mashhad	Shiraz	Yazd
Kurtosis	0.75	0.43	− 0.06	− 0.71	− 0.07	− 0.92
Slowness	− 0.70	− 0.84	− 0.51	− 0.37	− 1.1	− 0.76

In negatively Skewness, the mean of the data is less than the median. Negatively Skewness distribution is a type of distribution where the mean, median, and mode of the distribution are negative rather than positive or zero. Kurtosis is a statistical measure, whether the data is heavy-tailed or light-tailed in a normal distribution. Kurtosis less than 3 having a lower tail and stretched around center tails means most of the data points are present in high proximity with mean. A Kurtosis less than 3 distribution is flatter (less peaked) when compared with the normal distribution.

### Evaluation of solar radiation (R_s_) estimation models

In the studied stations, the values of R^2^, RMSE, and MBE for the calibrated models showed in Table 5. When tested using the R^2^ value, the calibrated models found to execute best in Mashhad, followed by Esfahan, Shiraz, Yazd, Kerman, and Bandar Abbas. Due to the inaccuracy in recording and many discarded data in the Bandar Abbas station, this station did not have very good results compared to other stations. The RMSE performance indicated that the calibrated models had the smallest error in Mashhad, followed by Esfahan, Bandar Abbas, Kerman, Shiraz, and Yazd. The mean RMSE values for the three improved models were lower than 1.3, which also indicated acceptable exactitude. The mean R^2^ value of the improved models was largest in Mashhad (0.977), followed by the values for Esfahan, Shiraz, Yazd, Kerman, and Bandar Abbas. The performance of the improved models in the same climates showed very small variation. The RMSE statistic showed that all models were more accurate in Esfahan, with an average value of 0.89 MJ m^−2^ day^−1^, followed by Bandar Abbas, Mashhad, Shiraz, Kerman, and Yazd. All improved models validated by the two statistical indicators performed well and that there was no significant difference between the models in each station and it shows that these two indicators could not be used alone to specify the best model in each station. Therefore, the MBE statistic used to determine the difference between the estimated data and measured data. Based on Performance indicators RMSE, MBE, calibration of the A–P model improved the accuracy of estimated R_s_ in most of the studied stations. If the value of R^2^ and RMSE are closer to one and zero respectively, the model is more appropriate.

**Table 5:** Statistical comparison of calibration (Ca) and validation (Va) estimated R_s_ (using the locally calibrated of the models coefficients).

Station	Algorithm		A–P model			Model 1			Model 2			Model 3		
			RMSE	R^2^	MBE	RMSE	R^2^	MBE	RMSE	R^2^	MBE	RMSE	R^2^	MBE
Bandar Abbas	SCE	Ca	1.13	0.841	0	1.41	0.841	− 0.80	1.13	0.841	0.00	1.17	0.840	0.30
		Va	1.60	0.835	− 0.41	2.08	0.840	− 1.25	1.60	0.835	− 0.41	1.55	0.836	− 0.11
	HS	Ca	1.16	0.835	0.21	1.16	0.835	− 0.03	1.22	0.816	− 0.01	1.25	0.823	− 0.16
		Va	1.53	0.836	− 0.19	1.62	0.827	− 0.43	1.69	0.807	− 0.42	1.63	0.818	− 0.52
	IHS	Ca	1.15	0.839	− 0.13	1.17	0.838	− 0.23	1.18	0.833	0.15	1.20	0.821	0.08
		Va	1.69	0.832	− 0.55	1.66	0.838	− 0.64	1.56	0.830	− 0.26	1.70	0.809	− 0.34
	GHS	Ca	1.16	0.841	− 0.19	1.17	0.841	− 0.16	1.14	0.840	− 0.09	1.35	0.825	0.62
		Va	1.61	0.839	− 0.58	1.56	0.841	− 0.54	1.62	0.835	− 0.49	1.73	0.814	0.16
Esfahan	SCE	Ca	0.83	0.970	0.09	0.83	0.970	0.01	0.83	0.970	0.09	0.83	0.969	0.01
		Va	1.3	0.941	0.40	1.29	0.940	0.32	1.31	0.940	0.4	1.29	0.946	0.32
	HS	Ca	0.84	0.962	− 0.07	0.90	0.966	− 0.19	0.96	0.964	− 0.02	1.13	0.943	0.12
		Va	1.26	0.940	0.24	1.29	0.937	0.14	1.40	0.935	0.26	1.53	0.923	0.36
	IHS	Ca	0.85	0.966	− 0.04	0.92	0.970	0.04	0.93	0.967	0.06	0.85	0.968	0.07
		Va	1.3	0.940	0.27	1.32	0.940	0.36	1.40	0.937	0.37	1.33	0.945	0.38
	GHS	Ca	0.84	0.968	− 0.12	0.83	0.970	0.01	0.87	0.970	− 0.28	0.94	0.968	0.26
		Va	1.27	0.941	0.19	1.28	0.940	0.32	1.24	0.940	0.03	1.39	0.946	0.58
Kerman	SCE	Ca	1.15	0.923	− 0.82	1.15	0.924	0.01	1.15	0.924	− 0.13	1.14	0.925	− 0.10
		Va	1.56	0.909	− 0.27	1.54	0.910	− 0.30	1.58	0.910	− 0.43	1.55	0.911	− 0.41
	HS	Ca	1.39	0.908	− 1.34	1.36	0.895	− 0.17	1.74	0.891	1.01	1.71	0.904	− 0.34
		Va	1.23	0.895	− 1.62	1.85	0.866	− 0.44	1.79	0.870	0.69	1.72	0.890	− 0.56
	IHS	Ca	1.22	0.908	− 1.10	1.24	0.923	0.18	1.30	0.912	0.18	1.29	0.917	− 0.21
		Va	1.26	0.923	− 1.35	1.71	0.910	− 0.17	1.74	0.897	− 0.13	1.55	0.901	− 0.50
	GHS	Ca	1.29	0.923	− 1.32	1.15	0.924	0.10	1.16	0.923	− 0.08	1.21	0.919	0.25
		Va	1.56	0.909	− 1.59	1.55	0.910	− 0.22	1.57	0.908	− 0.38	1.50	0.907	− 0.06
Mashhad	SCE	Ca	0.82	0.981	0.07	0.82	0.981	0.05	0.84	0.981	0.18	0.82	0.981	− 0.10
		Va	1.24	0.961	0.07	1.24	0.960	0.08	1.26	0.961	0.17	1.25	0.961	− 0.11
	HS	Ca	0.86	0.980	0.12	1.03	0.971	0.12	1.05	0.970	− 0.10	1.02	0.972	− 0.06
		Va	1.31	0.960	0.10	1.43	0.951	0.09	1.45	0.948	− 0.10	1.34	0.952	− 0.08
	IHS	Ca	0.84	0.981	0.18	0.90	0.977	− 0.05	0.88	0.979	0.14	1.03	0.976	− 0.12
		Va	1.26	0.960	0.17	1.30	0.957	− 0.05	1.27	0.959	0.12	1.30	0.957	− 0.12
	GHS	Ca	0.83	0.981	− 0.04	0.86	0.979	0.03	0.87	0.981	− 0.15	0.92	0.979	− 0.22
		Va	1.22	0.961	− 0.04	1.27	0.959	0.03	1.32	0.960	− 0.17	1.25	0.959	− 0.22
Shiraz	SCE	Ca	1.30	0.923	0.05	1.31	0.921	− 0.18	1.28	0.923	0.05	1.27	0.923	− 0.04
		Va	2.61	0.913	− 2.09	2.21	0.913	− 1.5	1.91	0.915	− 1.03	1.95	0.916	− 1.12
	HS	Ca	1.35	0.922	− 0.32	2.15	0.918	− 3.9	1.39	0.908	− 0.03	1.48	0.911	0
		Va	2.99	0.912	− 2.50	5.38	0.909	− 5.09	2.15	0.899	− 1.27	1.82	0.904	− 0.86
	IHS	Ca	1.32	0.922	0.20	1.40	0.917	0.45	1.35	0.917	0.02	1.38	0.913	− 0.11
		Va	2.54	0.912	− 1.97	1.90	0.912	− 0.75	1.83	0.909	− 0.91	2.04	0.900	− 1.16
	GHS	Ca	1.38	0.923	− 0.33	1.30	0.921	0.15	1.37	0.922	0.06	1.4	0.917	− 0.21
		Va	2.85	0.913	− 2.41	1.90	0.913	− 0.98	1.79	0.915	− 0.96	2.28	0.913	− 1.39
Yazd	SCE	Ca	2.67	0.921	− 2.36	2.21	0.921	− 2.65	1.51	0.924	0.04	1.50	0.925	0.02
		Va	2.39	0.916	− 2.68	2.11	0.916	− 2.33	1.72	0.919	0.48	1.71	0.920	0.51
	HS	Ca	2.03	0.920	− 1.39	1.73	0.913	− 0.30	1.58	0.919	0.15	1.69	0.904	− 0.04
		Va	1.75	0.913	0.36	1.99	0.910	0.81	1.78	0.918	0.55	1.86	0.897	0.20
	IHS	Ca	1.94	0.921	− 1.25	1.55	0.920	− 0.05	1.70	0.920	− 0.24	1.73	0.905	0.16
		Va	1.76	0.914	0.52	1.72	0.914	0.32	1.75	0.915	0.30	1.99	0.899	0.50
	GHS	Ca	2.00	0.921	− 1.32	1.56	0.922	− 0.26	1.56	0.924	0.25	1.59	0.920	0.19
		Va	1.73	0.915	0.45	1.66	0.917	0.19	1.84	0.919	0.67	1.88	0.915	0.73

RMSE (MJ m^−2^ day^−1^), MBE (MJ m^−2^ day^−1^).

### Comparison of results with other researchers

Calibrated the coefficients of the A–P model by various researchers shown in Table 6. In this research, the coefficients ‘a’ and ‘b’ calculated for the selected stations with different optimization algorithms (Table 3). Coefficient ‘a’ varies from 0.13 to 0.39, Also coefficient ‘b’ varies from 0.33 to 0.67 for six stations.

**Table 6:** Comparison of calibrated coefficients of the A–P model in the present study with the results of other researchers.

Station		Bandar Abbas		Esfahan		Shiraz		Kerman		Mashhad		Yazd	
		a	b	a	b	a	b	a	b	a	b	a	b
Khalili and Rezaei Sadr^50^				0.30	0.42	0.29	0.42	0.28	0.45	0.30	0.37	0.21	0.64
Sabziparvar et al.^49^				0.271	0.48	0.247	0.512	0.267	0.518	0.274	0.418	0.304	0.492
Didari and Ahmadi^51^						0.31	0.48						
Present study	SCE	0.38	0.35	0.15	0.58	0.25	0.53	0.27	0.51	0.22	0.62	0.18	0.53
	HS	0.38	0.36	0.13	0.60	0.26	0.50	0.28	0.47	0.24	0.59	0.20	0.64
	IHS	0.39	0.33	0.16	0.56	0.27	0.51	0.24	0.54	0.23	0.61	0.19	0.66
	GHS	0.36	0.37	0.15	0.57	0.20	0.58	0.26	0.50	0.21	0.63	0.18	0.67

In comparison with previous research, some differences observed between the results of this research and other works. For example, Sabziparvar et al.^49^, and Khalili and Rezaei Sadr^50^ applied the A–P model for Shiraz and reported the following pairs of ‘a’ and ‘b’, 0.247, 0.512; 0.29, 0.42, respectively While in the present research values of ‘a’ and ‘b’ coefficients are obtained as 0.25 and 0.53 with the SCE optimization algorithm for the same station; that is in good agreement with the coefficients of Sabziparvar et al. In this research, the A–P coefficients ‘a’ and ‘b’ with the SCE optimization algorithm are obtained 0.22 and 0.62 for Mashhad, but Khalili and Rezaei Sadr^50^, and Sabziparvar et al.^49^ reported, 0.30, 0.37 and 0.274, 0.418 for the same station, respectively. Sabziparvar et al.^49^, and Khalili and Rezaei Sadr^50^ suggested the application of the A–P model for the Esfahan station with the following pairs of coefficients ‘a’ and ‘b’: 0.271, 0.482; and 0.30, 0.42; but this research suggests values of 0.15 and 0.58 for ‘a’ and ‘b’ with the SCE optimization algorithm, respectively (Table 3). The inconsistent of the results can explained by a longer period of estimated R_s_, which applied in this research. Based on Liu et al.^23^, sample size and the length of the observation period could illustrate such differences in different researches. In addition, the rules for quality control of the R_s_ dataset and the higher restrictions for removing unreliable R_s_ data might somewhat cause such discrepancies (Table 6).

**Figure 2:**
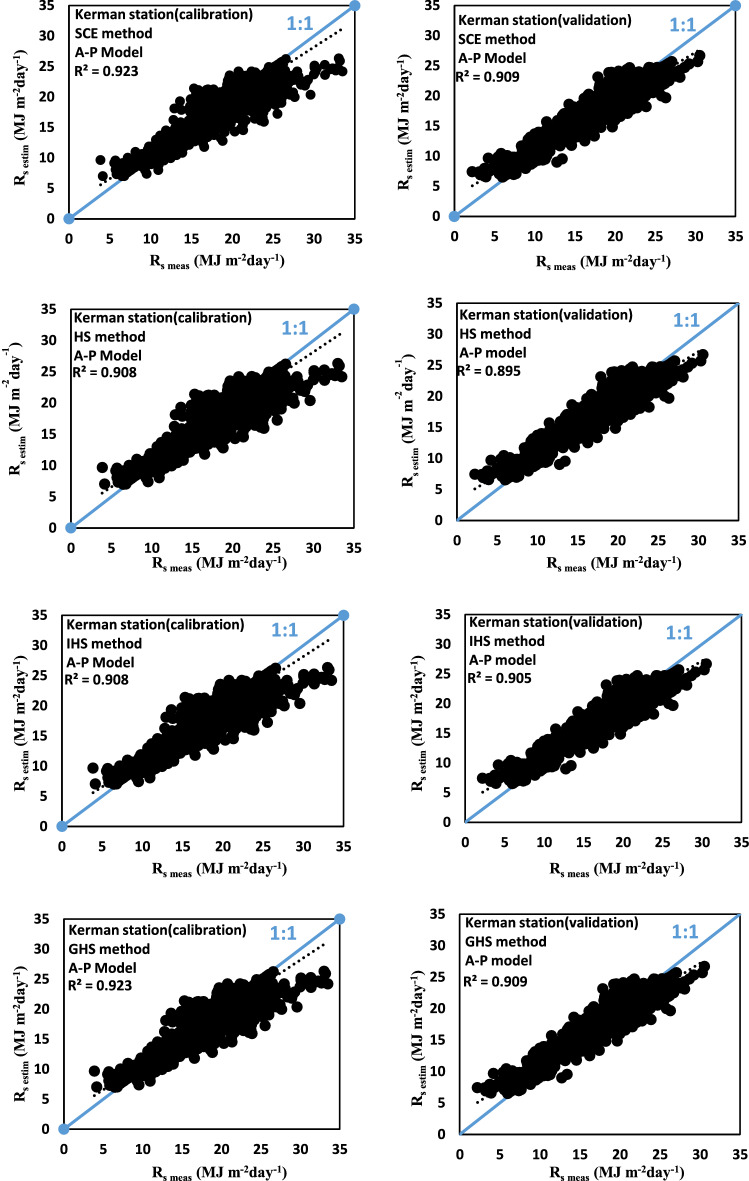
Comparison of measured and estimated Rs in the A–P model.

The values of measured and estimated global solar radiation are compared by the A–P model from 1992 to 2017 as shown in Fig. 2. To appraise the prediction accuracy of R_s_, computed from the regional best performing estimated data and the measured data, specific values of the A–P model statistics by different optimization algorithms (HS, IHS, GHS, and SCE) compared in the Kerman station. In addition, the R^2^ values of both the measured data and the estimated data in this station were very close to the 1:1 line, which means that the R_s_ determined from the estimated data and measured data were in good accordance.

According to Table 5 and Fig. 2, the calibration and validation performance of the A–P model were better than the three improved models in all stations. As shown in Table 5, the RMSE varies between 0.82 and 2.67 MJ m^−2^ day^−1^ for the A–P model with the SCE algorithm in the calibration phase. Besides, other indicators were lower in the case of the A–P models in the SCE algorithm. Based on the results in Tables 5 and 6, the decrease rate of RMSE values in various stations for four optimization algorithms was different. For example, in the SCE algorithm, the value of RMSE decreased by about 4% and 7% for Mashhad and Kerman stations in the calibration phase contrasted to the HS algorithm, respectively. In other words, the highest decrease of RMSE related to the Kerman station. The lowest value of R^2^ is observed in the Bandar Abbas station (R^2^ = 0.81). Further, according to MBE values, a decrease occurred in the MBE of all stations in the SCE algorithm contrasted to three algorithms (IHS, GHS, and HS), in the A–P and three improved models.

**Figure 3:**
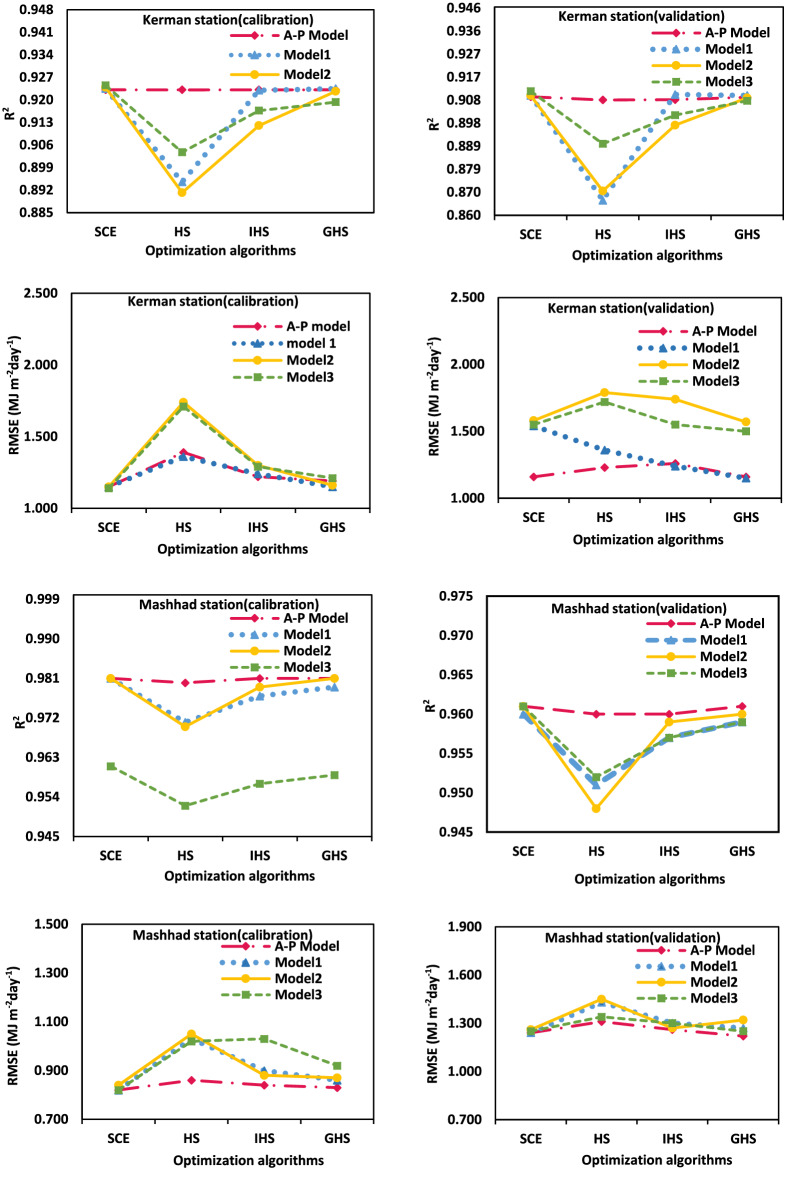
Comparison R^2^ and RMSE between the calibrated and validation model with different optimization algorithms for Mashhad and Kerman stations.

The values of R^2^ and RMSE for Mashhad and Kerman stations by different optimization algorithms, the A–P model, and the three improved models is shown in Fig. 3.

**Figure 4:**
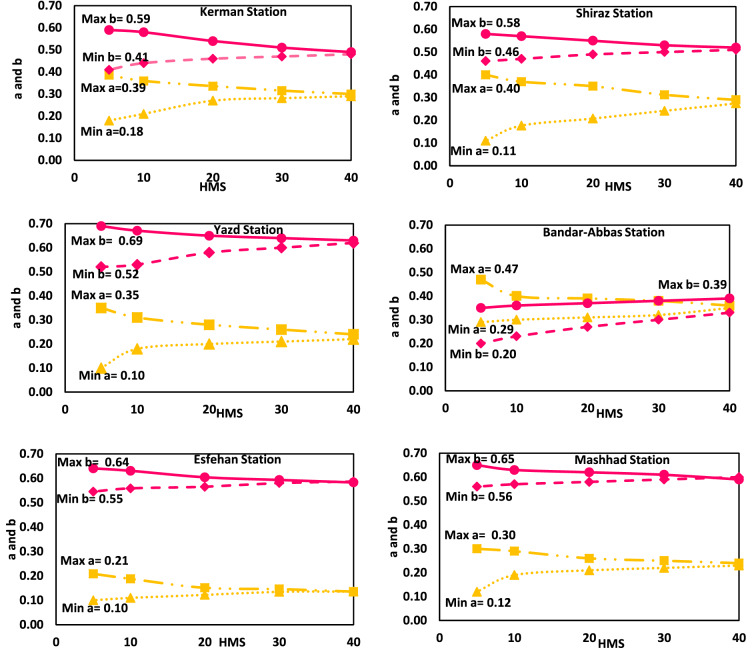
The minimum and maximum A–P model coefficients in Hs method, in different harmony memory size (HMS).

The values of ‘a’ and ‘b’ in the harmonic memory sizes (HMS) (5, 10, 20, 30, and 40) are shown in six meteorological stations in Fig. 4. This Figure shows that as the initial population increases, the values of the coefficients become convergent and a smaller range for the coefficients obtain in different stations. For example, in the Kerman station, with increasing HMS, the minimum and maximum coefficient ‘a’, changes from 0.18 to 0.35 and from 0.39 to 0.36, respectively. The maximum and minimum values of ‘a’ are close to each other, which is true for coefficient ‘b’.

## Conclusion

In this article, Harmony Search (HS), Global Harmony Search (GHS), Improved Harmony Search (IHS), and Shuffled Complex Evolution (SCE) optimization algorithms were used to calibrate the coefficients of the R_s_ model and its three improved models (on the six meteorological stations in Iran from 1992 to 2017). For practical usage, using a calibrated form of the A–P model seems necessary for Iran’s climatic situations.

Coefficients of models in which the T and RH used calibrate by optimization methods. The results showed that adding T_max_, T_min,_ and RH_mean_ did not affect the A–P model. In addition, the SCE optimization algorithm method has shown better results than other optimization methods. Table 7 presents the final models for the studied stations.

**Table 7:** Original equations obtained in this research for the estimation of solar radiation by SCE algorithm.

Station	A–P model	R^2^	Model 3	R^2^
Bandar Abbas	R_s_ = ( 0.38 + 0.35 × (n/N)) × R_a_	0.841	R_s_ = (0.40 + (0.35 × (n/N)) − 0.0019 × (T_max_ − T_min_) + 0.000 × (RH_mean_)) × R_a_	0.840
Esfahan	R_s_ = (0.15 + 0.58 × (n/N)) × R_a_	0.970	R_s_ = (0.15 + (0.58 × (n/N)) − 0.0004 × (T_max_ − T_min_) + 0.000 × (RH_mean_)) × R_a_	0.969
Kerman	R_s_ = (0.27 + 0.51 × (n/N)) × R_a_	0.923	R_s_ = (0.29 + (0. 50 × (n/N)) − 0.0019 × (T_max_ − T_min_) − 0.0003 × (RH_mean_)) × R_a_	0.925
Mashhad	R_s_ = (0.22 + 0.62 × (n/N)) × R_a_	0.981	R_s_ = (0.23 + (0.61 × (n/N)) − 0.0007 × (T_max_ − T_min_) − 0.0001 × (RH_mean_)) × R_a_	0.981
Shiraz	R_s_ = (0.25 + 0.53 × (n/N)) × R_a_	0.923	R_s_ = (0.30 + (0.51 × (n/N)) − 0.0012 × (T_max_ − T_min_) − 0.0007 × (RH_mean_)) × R_a_	0.923
Yazd	R_s_ = (0.18 + 0.53 × (n/N)) × R_a_	0.921	R_s_ = (0.24 + (0.65 × (n/N)) − 0.0035 × (T_max_ − T_min_) − 0.0007 × (RH_mean_)) × R_a_	0.925

Considering the sunshine, which is an important factor for estimating R_s_, and accepting that Iran is a country in which sunshine is significant, the Angstrom empirical model can well estimate total radiation. The coefficients ‘a’ and ‘b’ have calibrated in this research. Coefficient ‘a’ varies from 0.1 to 0.47 and coefficient ‘b’ varies from 0.2 to 0.69 for studied stations.

In this research, the three R_s_ estimation models have appraised and calibrated. The results indicate that the A–P model (R^2^ = 0.981 in Mashhad station) offers the best R_s_ estimations in the semi-arid and arid climate among the improved models, as compared to the measured R_s_.
